# Dependence of lumbar loads on spinopelvic sagittal alignment: An evaluation based on musculoskeletal modeling

**DOI:** 10.1371/journal.pone.0207997

**Published:** 2019-03-18

**Authors:** Tito Bassani, Gloria Casaroli, Fabio Galbusera

**Affiliations:** IRCCS Istituto Ortopedico Galeazzi, Milan, Italy; Universidad de Zaragoza, SPAIN

## Abstract

Still little is known about how spinopelvic alignment affects spinal load distribution. Musculoskeletal modeling can potentially help to discover associations between spine alignment and risk factors of spinal disorders (e.g. disc herniation, vertebral fracture, spondylolisthesis, low back pain). The present study exploited the AnyBody full-body musculoskeletal model to assess the relation between lumbar loads and spinopelvic alignment in the sagittal plane. The model was evaluated in the standing position. The simulated postures were set using spinopelvic parameters gleaned from the literature and characterizing the healthy adult population. The parameters were: sagittal vertical axis, Roussouly lumbar type, sacral slope, and pelvic incidence. A total of 2772 configurations were simulated based on the following measurements: compression force and anterior shear at levels L4L5 and L5S1; multifidus, longissimus spinae, and rectus abdominis muscle forces. Changes in global sagittal alignment, lumbar typology, and sacral inclination, but not in pelvic incidence, were found to affect intervertebral loads in the lumbar spine and spinal muscle activation. Considering these changes would be advantageous for clinical evaluation, due to the recognized relation between altered loads and risk of disc herniation, vertebral fracture, spondylolisthesis, and low back pain. Musculoskeletal modeling proved to be a valuable biomechanical tool to non-invasively investigate the relation between internal loads and anatomical parameters.

## Introduction

The human spine forms an S-shape in the sagittal plane, with convex curvature in the thoracic and sacral regions. The anatomical spinopelvic parameters, obtained by radiographic examination and clinically used to describe spine alignment, are related to four spine regions: cervical, thoracic, lumbar, and sacropelvic.

The most common parameter to assess global alignment is the sagittal vertical axis (SVA), which is defined as the horizontal offset from a plumb line dropped from the seventh cervical vertebra to the posterior-superior corner of the sacral endplate [1]. Being an index of sagittal imbalance, when the SVA magnitude exceeds the normal range, either toward the front or the back, the spine is considered malaligned. The lumbar spine is lordotic in shape (i.e., convex anteriorly). However, although identified as lordotic, this section presents different patterns within the healthy population and is classified according to four Roussouly types (RT) [2]. The RT parameter differentiates the lumbar spine by the vertebral level of curve apex and the degree of sacral inclination. In the sacropelvic region, the interrelationship between sacral slope (SS), pelvic incidence (PI), and pelvic tilt (PT) angles is expressed with the geometrical formula: PI = SS+PT [3,4]. PI defines the relative orientation of the sacrum versus the ilium, and SS and PT are dynamic parameters that change as the pelvis rotates about the hip axis.

Biomechanically, spinopelvic parameters are expected to be associated with spinal loads and muscle activation [5–8]. Changes in these biomechanical measures in maintaining posture or performing movements can affect the health status of the spinal musculoskeletal system. Accordingly, spinopelvic parameters were found related to quality of life outcomes [9–11]. A significant correlation exists between pelvic retroversion associated with loss of lumbar lordosis and quality of life scores [12,13]. Even mild spine malalignment can be detrimental [14]. Since spinopelvic parameters play a major role in determining disability in adults with spinal deformity [12,14,15], target values of sagittal spinopelvic alignment for satisfactory outcomes after spinal reconstruction have been proposed [13]. Moreover, the importance of spinopelvic balance and its implications for the clinical treatment of low back pain have been demonstrated [16,17].

Yet little is known about how spinopelvic alignment affects spinal load distribution. Musculoskeletal modeling can potentially help to discover associations between spine alignment and the development of risk factors of spinal disorders (e.g., disc herniation, vertebral fracture, spondylolisthesis, low back pain). Unfortunately, assessing the relation between biomechanical loads and spinopelvic parameters based on *in vivo* measurements is unfeasible. Indeed, acquiring internal loads is highly invasive and identifying the anatomical parameters requires radiographic examination. Conversely, musculoskeletal modeling allows non-invasive investigation of this relation via the so-called inverse dynamic approach, which provides loads and muscle activation in assigned postures or movements. To date, no previous studies have extensively investigated the relation between spinal loads and spinopelvic parameters in physiological conditions. Sasaki et al. [18] expanded their previously validated model [19] to evaluate sagittal parameters and kinematic measurements at the hip and the knee joint during standing and walking in elderly women with pelvic retroversion. Senteler et al. [20] investigated intervertebral forces in relation to the mismatch between pelvic incidence and lumbar lordosis in patients with lumbar fusion. Using a patient-specific rigid body model (in OpenSim software) based on preoperative radiographs, the authors found higher shear forces in alignments considered to be at risk for adjacent segment disease. Moreover, by exploiting OpenSim, Bruno et al. [21] have recently shown that the prediction of vertebral loading is dependent on subject-specificity of spinal curvature and muscle morphology.

Here, we employed the AnyBody full-body musculoskeletal model to assess the changes in lumbar load (i.e., intersegmental forces and spinal muscle activation) in relation to physiological changes in four spinopelvic parameters (SVA, RT, SS, and PI). The ranges for these parameters were taken from the literature. Since they were originally acquired from radiographic examination of upright subjects, the parameters were used to generate corresponding arrangements of standing postures with the AnyBody model.

## Methods

### Musculoskeletal model

**Fig 1:**
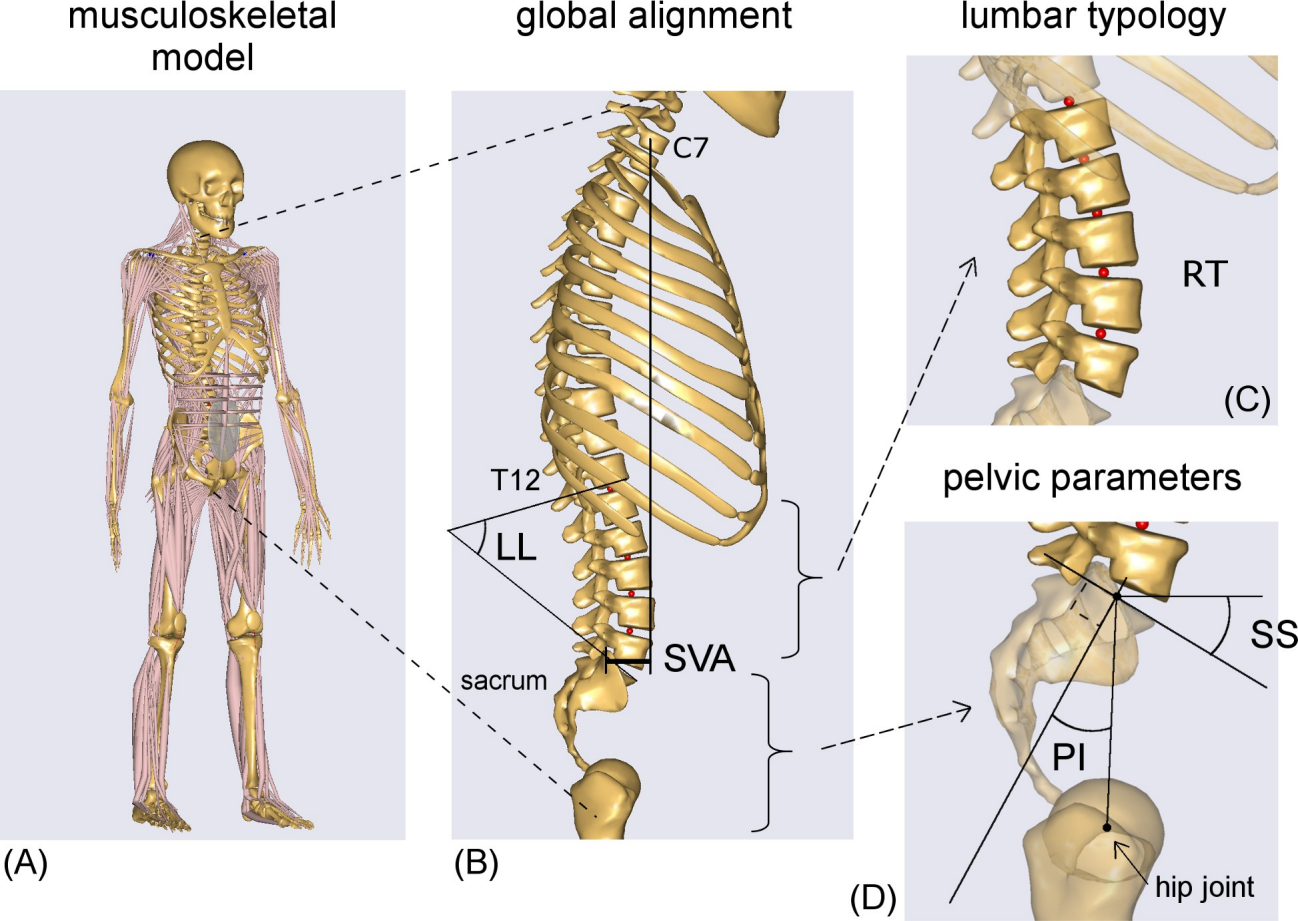
Full-body model and anatomical parameters. The AnyBody full-body musculoskeletal model in standing position (A) and sagittal views depicting sagittal vertical axis (SVA) (B), Roussouly lumbar typology (RT) (C), sacral slope (SS), and pelvic incidence (PI) (D). Muscles, arms, and pelvis are not shown in the sagittal views.

The full-body musculoskeletal model from the AnyBody Managed Model Repository (AMMR, v.1.6.3) with AnyBody software v.6.1 (AnyBody Technology, Denmark) was used for the simulations (Fig 1A). By default, the body model represents the size and weight of an average European male (1.76 m, 75 kg). The model has been extensively validated for the assessment of lumbar loads and muscle activation during assigned postures and movements in physiological conditions [22–24]. Musculoskeletal modeling characterizes bones as rigid segments connected by joints, and muscles as tensile elements attached to segments and providing movements. By means of the inverse dynamic approach, muscle forces and intersegmental forces acting during the execution of specific imposed kinematics are computed by minimizing muscle recruitment activation [25,26]. The default polynomial optimization criterion was exploited. For the spine, the model characterizes the twelve thoracic vertebrae and ribcage as a single segment, the five lumbar vertebrae (from L1 to L5) as segments connected by spherical joints, and the sacrum and pelvis as rigidly connected segments. A total of 188 muscle fascicles and abdominal pressure are accounted for in the lumbar region.

### Simulation process

**Fig 2:**
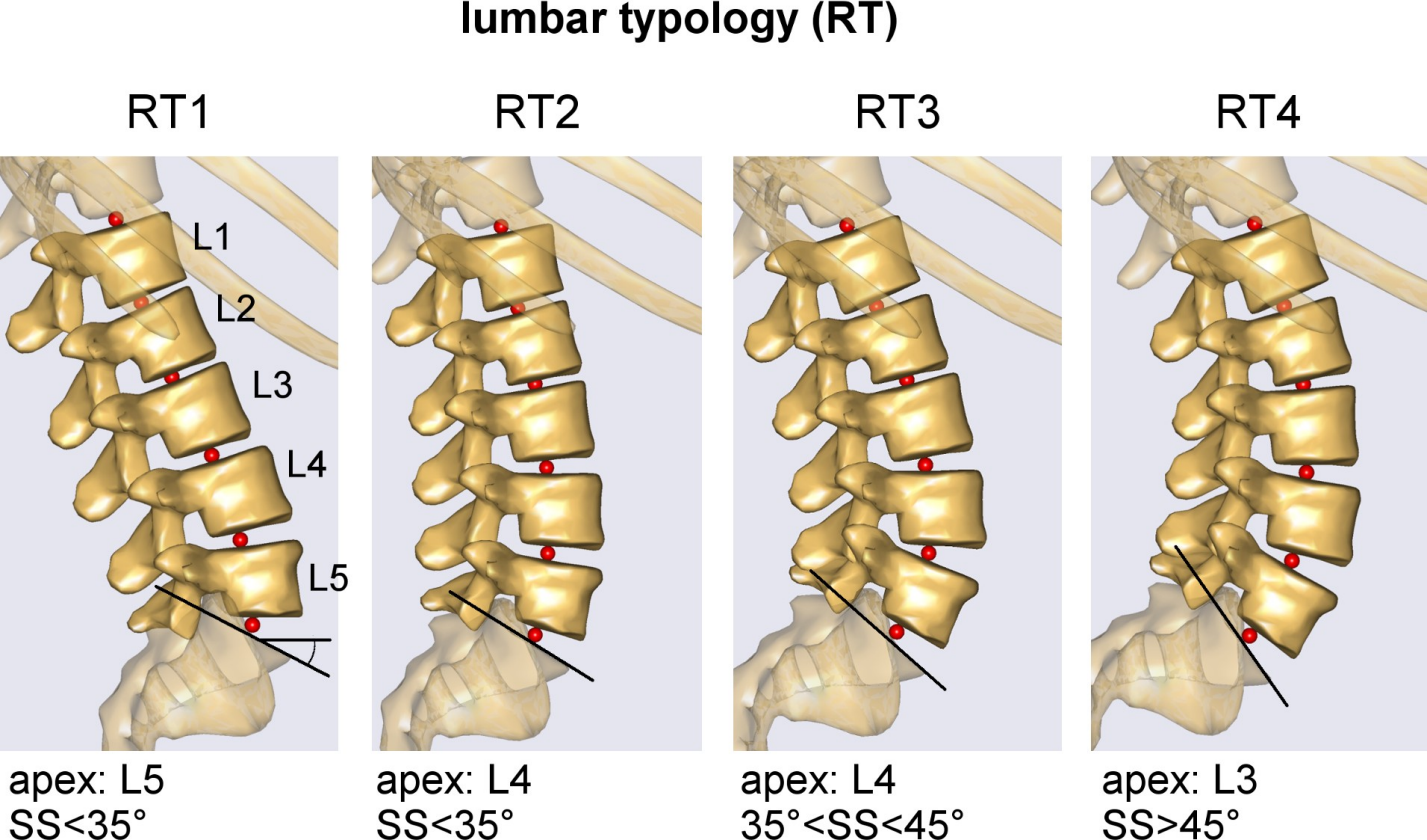
Modeling of the lumbar typologies. Modeling of the alignment of the lumbar vertebrae (from L1 to L5) and intervertebral joints (depicted as red spheres) in the four Roussouly types (RT) [2]. Curve apex and sacral slope (SS) identifying the different RTs are reported.

The full-body model was evaluated in standing postures simulating spinopelvic alignment (Fig 1) corresponding to changes in four anatomical parameters (SS, PI, RT, and SVA) in asymptomatic adults. Since RT implicitly accounts for changes in lumbar lordosis (LL), LL was not included as an additional parameter in this study. However, the corresponding LL values for the four different RTs are given in the results. The parameters values (Table 1) were obtained from the study by Hu et al. [27] who provided the mean values and the ranges of SS, PI, and SVA differentiated in the four RTs (Fig 2). Increments of 1° were considered for SS and PI. Model posture was set by adjusting the anatomical parameters in the following sequence: SS, PI, RT, and SVA.

**Table 1:** Anatomical parameters and reference rotations of the lumbar vertebrae.

	SS [°]	PI [°]	SVA [cm]			vertebral rotation [°] (positive in flexion)				
			back	med	front	L1	L2	L3	L4	L5
**RT1**	25–35	30–50	-3.0	3.0	9.0	-17	-22	-27	-19	-8
**RT2**	25–35	30–50	-4.9	1.1	7.1	-12	-13	-8	-1	9
**RT3**	35–45	40–60	-4.5	1.5	7.5	-20	-22	-12	-2	22
**RT4**	45–55	50–70	-6.3	-0.3	6.3	-20	-14	-2	9	30

Range of pelvic parameters (sacral slope (SS) and pelvic incidence (PI)) and sagittal vertical axis (SVA, in backward, medium, and frontward alignment) for the four Roussouly lumbar types (RT) taken from Hu et al. [27] and used in the simulations. Reference vertebral orientations in the RT were obtained by manually measuring the examples reported in Roussouly et al. [2].

**Fig 3:**
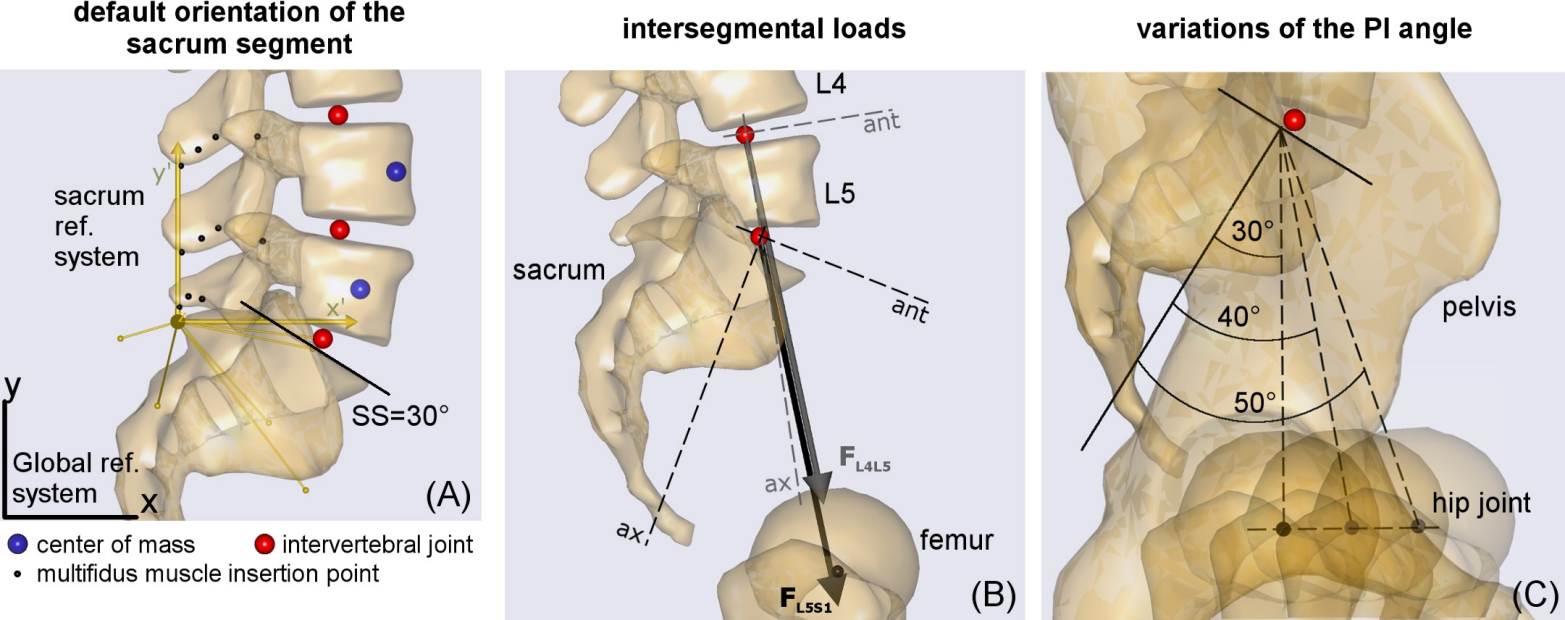
Sacrum segment orientation, intersegmental load, and PI changes. Panel (A): default orientation of the sacrum segment and mesh surfaces of the sacrum and lower lumbar vertebrae. The sacrum local reference system (x',y') with reference nodes is given in yellow and the global reference system (x,y) in black. The sacral slope (SS) is obtained by manually measuring the endplate inclination from the view. Panel (B): example of predicted intersegmental loads at levels L4L5 and L5S1 (F_L4L5_ and F_L5S1_), and corresponding axial and anterior directions. The configuration is RT1, with SS 25°, PI 40°, and SVAmed. Panel (C): changes in the PI angles in the assigned RT and SS.

By default, the local reference system for the sacrum segment is oriented parallel to the global reference system, but the SS is approximately 30° (Fig 3A). Accordingly, the changes in SS were simulated by rotating the sacrum segment to account for the SS default value. For example, the segment was rotated 10° anteriorly to simulate a SS of 40°. This movement also provided the corresponding rotations of the segment properties, such as joints and muscle insertion points, thus preserving the morphological characterization of the sacrum and the pelvis (the sacrum and pelvis being rigidly connected).

For the PI, changes in this angle were simulated by shifting, in the sagittal plane, the position of the hip joints (defined in the pelvis segment) that connect the pelvis to the femoral heads (Fig 1D and Fig 3C). This procedure changed the attachment point between the femur and the pelvis, without altering the position of the muscle insertion points in the respective segment. After setting the SS and the PI, the four RTs (RT1, RT2, RT3, and RT4) were modeled to match the reference examples reported by Roussouly et al. [2]. Each RT was obtained by rotating the vertebral segments in the sagittal plane (Table 1). Proceeding from L5 to L1, each vertebral segment was rotated to shift the slope of the vertebral mesh from its original value in the default AnyBody model (Fig 1C) to that required in the specific RT (Fig 2). With this approach, morphological characterization (i.e., position of the intervertebral joints, vertebral centroid, and muscle insertion points) is kept unaltered for each vertebra (Fig 3A). Furthermore, the position of the center of mass of the vertebral segments was placed more anteriorly than the vertebral centroids, depending on the lumbar level, according to the computed tomography (CT) scan measurements reported by Pearsall et al. [28] (Fig 3A).

Finally, the three SVA values (dependent on RT, Table 1) corresponding to balanced alignment (SVAmed) and to backward and frontward imbalanced postures (SVAback and SVAfront) were modelled. In detail, the required SVA was achieved by setting the rotation of the thoracic segment with respect to the L1 vertebra (Fig 1B). Neck rotation was set opposite to thoracic rotation in order to maintain horizontal gaze; arms were kept vertically aligned.

In all, 2772 spinopelvic configurations were simulated. The simulations were run in batch process using custom routines written in MATLAB (MathWorks Inc., Natick, MA, USA).

### Model outputs

The following measurements were computed for each simulated configuration: intersegmental force at L4L5 (F_L4L5_) and at L5sacrum (F_L5S1_) joints; muscle forces of the multifidus (F_MF_), longissimus spinae (F_LS_), and rectus abdominis (F_RA_), these muscles being involved in maintaining the spine aligned in upright posture. The axial compression and the anterior shear components of F_L4L5_ ($F_{L4L5}^{c}$ and $F_{L4L5}^{s}$) were obtained by projecting F_L4L5_ on the axis passing through the upper and lower intervertebral joints of L5 (axial direction, caudally oriented) and on the orthogonal axis (anteriorly oriented, parallel to the upper endplate), respectively (Fig 3B). The components of F_L5S1_ ($F_{L5S1}^{c}$ and $F_{L5S1}^{s}$) were obtained by projecting F_L5S1_ on the axis orthogonal to the direction of the default sacral inclination (axial direction, caudally oriented) and on the orthogonal axis (anteriorly oriented, parallel to the sacral endplate), respectively (Fig 3B).

## Results

### Intersegmental forces

**Fig 4:**
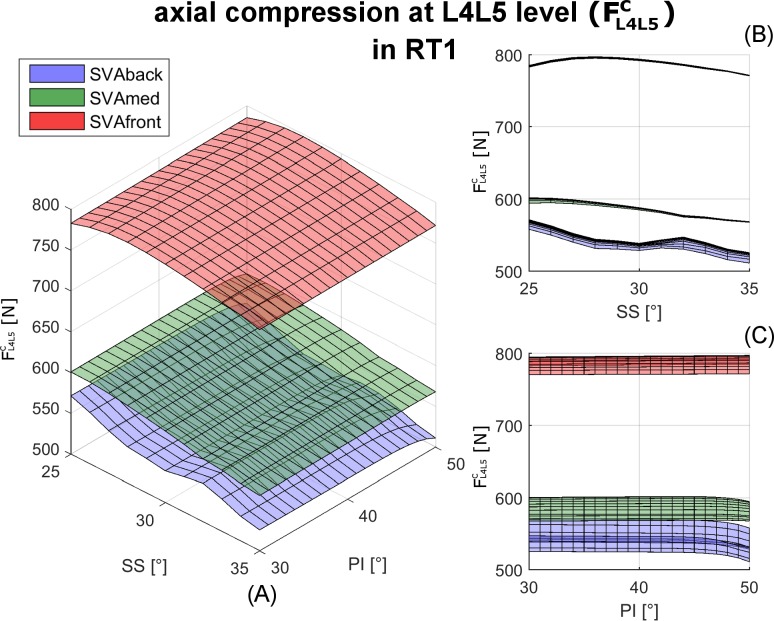
Results for F_c_ in RT1. Box (A): axial compression force at level L4L5 ($F_{L4L5}^{c}$), computed in relation to changes in sacral slope (SS) and pelvic incidence (PI) in RT1. The results for the three sagittal vertical axis (SVA) conditions interpreting the balanced global alignment (SVAmed) and backward and frontward imbalanced alignments (SVAback and SVAfront) are presented as green, blue, and red surfaces, respectively. The two boxes (B and C) on the right present the results in the SS and PI perspectives, respectively.

**Fig 5:**
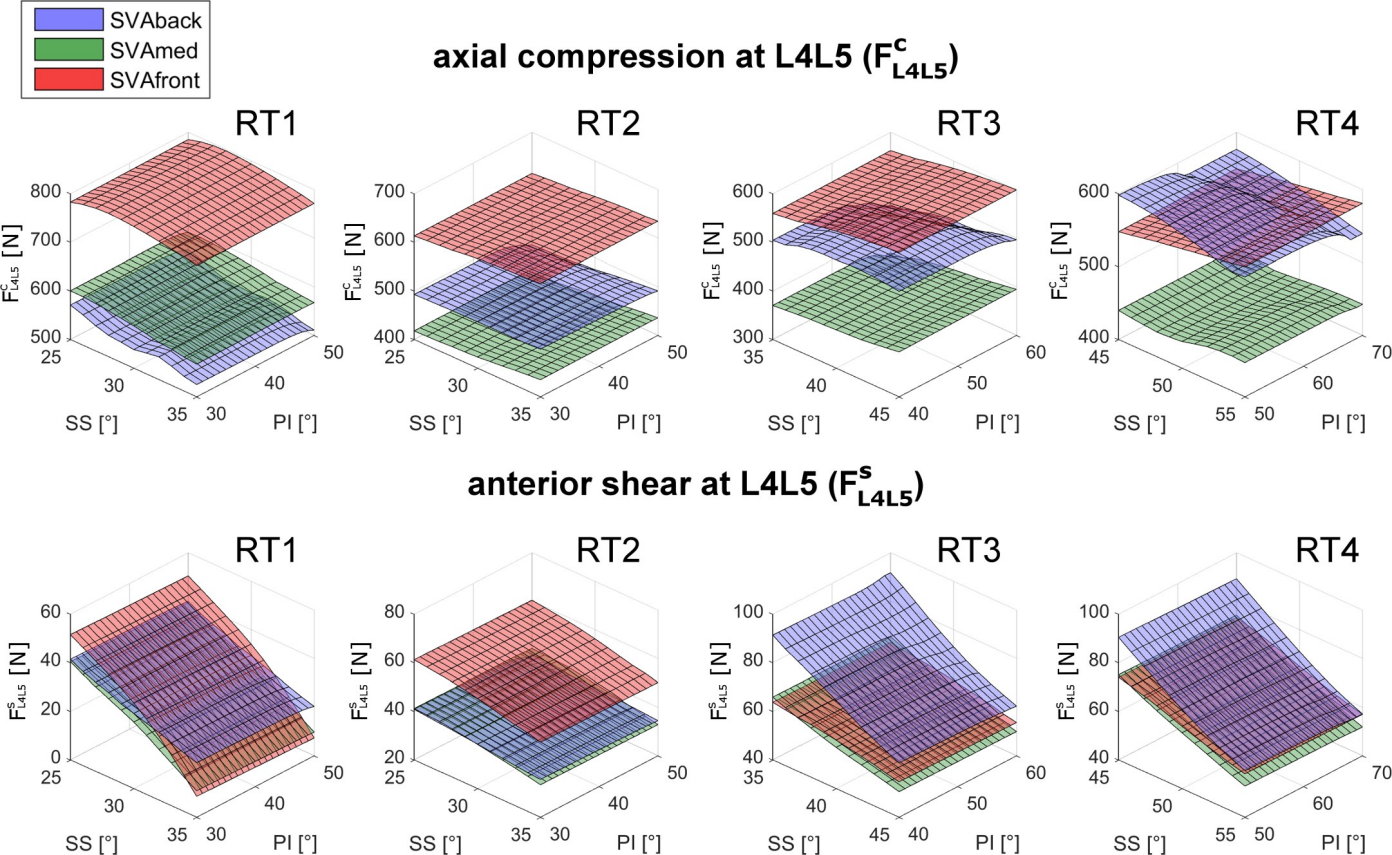
Results for $F_{L4L5}^{c}\;and\;F_{L4L5}^{s}$ in all four RTs. Axial compression force ($F_{L4L5}^{c}$, upper row) and anterior shear ($F_{L4L5}^{s}$, lower row) computed in relation to changes in sacral slope (SS) and pelvic incidence (PI) in the four lumbar typologies (RT1, RT2, RT3, RT4). The results for the three sagittal vertical axis (SVA) conditions interpreting the balanced global alignment (SVAmed) and the backward and frontward imbalanced alignments (SVAback and SVAfront) are presented as green, blue, and red surfaces, respectively.

**Fig 6:**
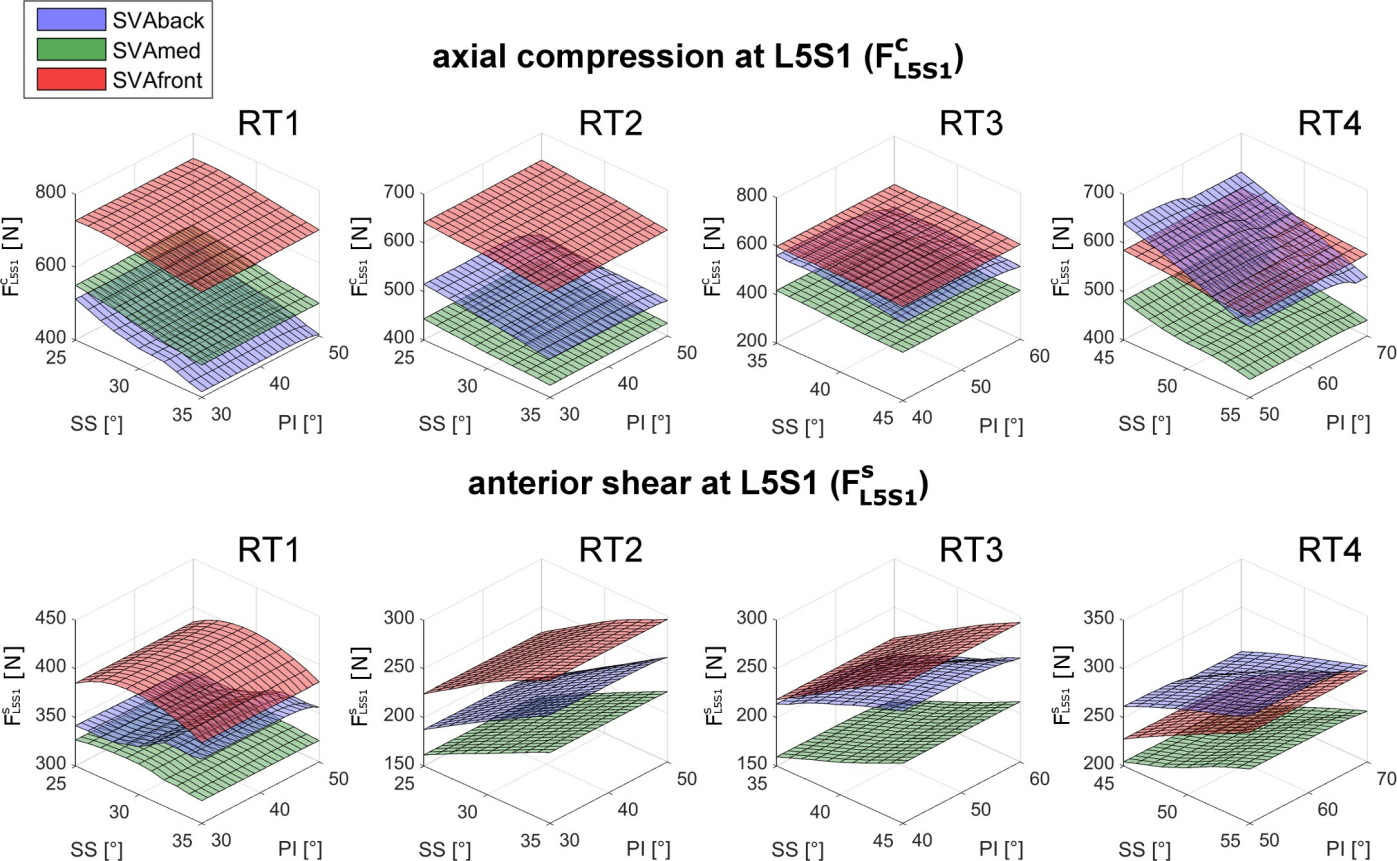
Results for $F_{L5S1}^{c}\;and\;F_{L5S1}^{s}$ in all four RTs. Axial compression force ($F_{L5S1}^{c}$, upper row) and anterior shear ($F_{L5S1}^{s}$, lower row) computed in relation to changes in sacral slope (SS) and pelvic incidence (PI) in the four lumbar typologies (RT1, RT2, RT3, RT4). The results for the three sagittal vertical axis (SVA) conditions interpreting the balanced global alignment (SVAmed) and the backward and frontward imbalanced alignments (SVAback and SVAfront) are presented as green, blue, and red surfaces, respectively.

The compression force at L4L5, $F_{L4L5}^{c}$, in RT1 was larger in SVAfront alignment than in SVAmed and SVAback (Fig 4A). A moderate negative linear relation was observed to depend on SS changes in SVAmed and SVAback (Fig 4B). Changes in PI did not substantially affect the $F_{L4L5}^{c}$ values (Fig 4C). This lack of relation with PI changes was, in general, evident in all the predicted compression and shear forces at both L4L5 and L5S1 (Figs 5 and 6). Generally, a balanced alignment posture (SVAmed) produced lower compression and shear forces (upper row in Figs 5 and 6). RT1 produced higher $F_{L4L5}^{c}$, lower $F_{L4L5}^{s}$, and higher $F_{L5S1}^{s}$ than the other RTs (Table 2). Since the forces were mainly arranged as planar surfaces (Figs 5 and 6), which are intrinsically characterized by a non-Gaussian distribution, the descriptive values are reported as median and range in Tables 2 and 3. In RT1 the median $F_{L4L5}^{c}$ ranged from 543N to 790N, the median $F_{L4L5}^{s}$ from 27N to 35N, and the median $F_{L5S1}^{s}$ from 329N to 398N. The ranges for RT3 and RT4 were larger, with a median $F_{L4L5}^{s}$ from 57N to 76N and from 63N to 72N, respectively.

**Table 2:** Predicted intersegmental forces.

			SVA		
			back	med	front
**level** **L4L5**	$F_{L4L5}^{c}$	RT1	543(525–570)	588(569–602)	790(771–796)
		RT2	496(493–498)	424(419–437)	626(614–635)
		RT3	520(502–524)	381(372–395)	584(561–599)
		RT4	581(563–589)	440(434–448)	565(549–580)
	$F_{L4L5}^{s}$	RT1	31(22–41)	27(10–40)	35(8–51)
		RT2	38(35–41)	38(33–41)	57(51–61)
		RT3	76(62–91)	57(50–66)	60(54–63)
		RT4	72(59–90)	63(53–75)	65(58–74)
**level** **L5S1**	$F_{L5S1}^{c}$	RT1	456(415–512)	520(490–552)	717(690–730)
		RT2	499(478–516)	433(426–445)	632(617–643)
		RT3	550(520–559)	408(401–419)	598(589–600)
		RT4	591(545–628)	453(437–481)	577(567–586)
	$F_{L5S1}^{s}$	RT1	349(341–366)	329(323–332)	398(383–403)
		RT2	226(187–261)	192(162–223)	265(224–297)
		RT3	242(213–265)	184(160–212)	258(219–294)
		RT4	285(258–310)	228(204–256)	262(228–294)

Median and range of the computed axial compression and anterior shear forces at level L4L5 ($F_{L4L5}^{c}$ and $F_{L4L5}^{s}$) and at L5S1 ($F_{L5S1}^{c}$ and $F_{L5S1}^{s}$), expressed in Newton. Values for the four Roussouly types (RT1, RT2, RT3, RT4) corresponding to changes in sagittal vertical axis (SVA, in backward, medium, and frontward alignment).

**Table 3:** Predicted muscle forces.

		SVA		
		back	med	front
**F**_**MF**_	RT1	16(11–21)	18(15–22)	41(27–48)
	RT2	18(17–18)	17(16–18)	24(23–25)
	RT3	31(26–37)	25(23–30)	42(29–51)
	RT4	28(25–32)	29(25–44)	48(36–57)
**F**_**ES**_	RT1	52(46–60)	150(140–156)	299(281–312)
	RT2	25(25–25)	65(62–70)	194(185–200)
	RT3	71(65–73)	83(76–87)	202(199–210)
	RT4	89(87–92)	97(96–98)	192(191–198)
**F**_**RA**_	RT1	26(21–33)	-	-
	RT2	85(84–85)	-	-
	RT3	123(115–129)	-	-
	RT4	175(167–183)	32(28–37)	-

Median and range of the computed muscle forces (F_MF_, F_ES_, F_RA_) expressed in Newton. Values for the four Roussouly types (RT1, RT2, RT3, RT4) corresponding to changes in sagittal vertical axis (SVA, in backward, medium, and frontward alignment). -, indicates that the muscle was not activated, thus producing null muscle force.

**Fig 7:**
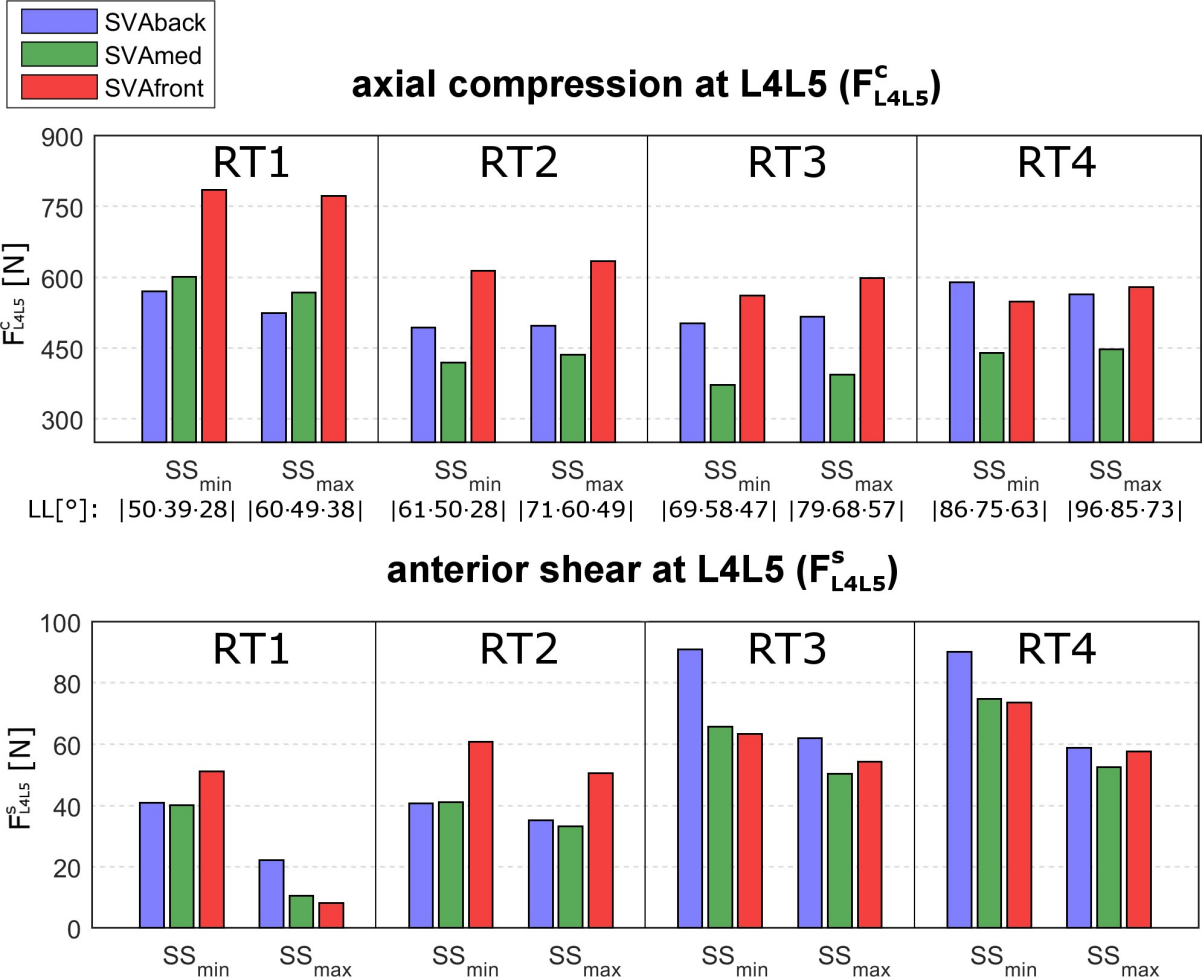
$F_{L4L5}^{c}\;and\;F_{L4L5}^{s}$ in relation to SS. Axial compression force ($F_{L4L5}^{c}$, upper plot) and anterior shear ($F_{L4L5}^{s}$, lower plot) at the L4L5 level, computed at minimum and maximum sacral slope (SS_min_ and SS_max_) for the four lumbar typologies (RT1, RT2, RT3, RT4). SS_min_ and SS_max_ were, respectively, 25° and 35° for both RT1 and RT2, 35° and 45° for RT3, and 45° and 55° for RT4 (Table 1). The results correspond to the central pelvic incidence (PI) values for the RT (40° in RT1 and RT2, 50° in RT3, 60° in RT4, see Table 1). The three sagittal vertical axis (SVA) conditions interpreting the balanced global alignment (SVAmed) and the backward and frontward imbalanced alignments (SVAback and SVAfront) are presented as green, blue, and red bars, respectively. The lumbar lordosis (LL) is reported as well.

**Fig 8:**
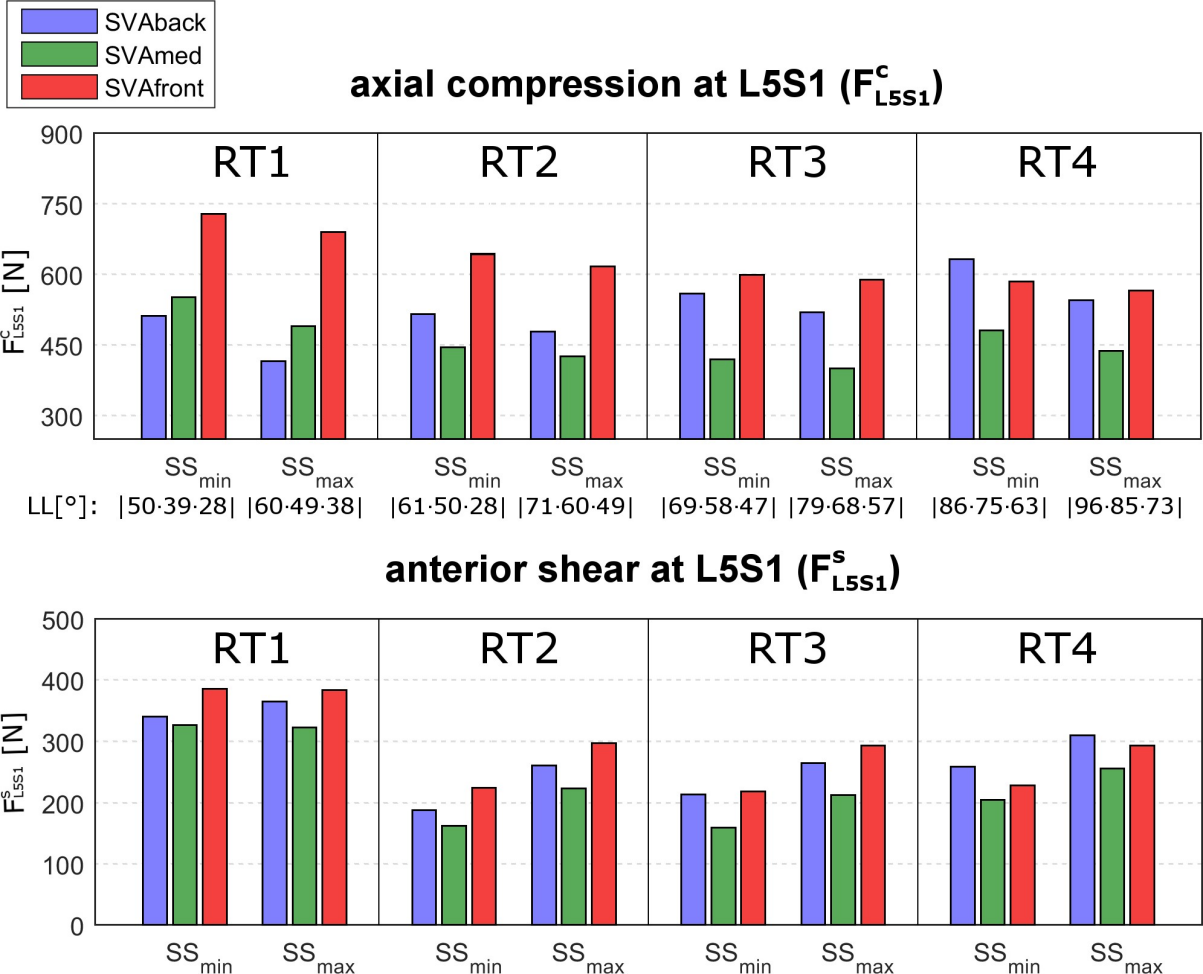
$F_{L5S1}^{c}\;and\;F_{L5S1}^{s}$ in relation to SS. Axial compression force ($F_{L5S1}^{c}$, upper plot) and anterior shear ($F_{L5S1}^{s}$, lower plot) at the L5S1 level computed at the minimum and maximum sacral slope values (SS_min_ and SS_max_) in the four lumbar typologies (RT1, RT2, RT3, RT4). SS_min_ and SS_max_ are, respectively, 25° and 35° for both RT1 and RT2, 35° and 45° for RT3, 45° and 55° for RT4 (Table 1). The results correspond to the central pelvic incidence (PI) values for the RT (40° in RT1 and RT2, 50° in RT3, 60° in RT4, see Table 1). The three sagittal vertical axis (SVA) conditions interpreting the balanced global alignment (SVAmed) and the backward and frontward imbalanced alignments (SVAback and SVAfront) are presented as green, blue, and red bars, respectively. The lumbar lordosis (LL) is reported as well.

In order to assess the dependence of the intersegmental forces on the changes in the SS, we compared the force values computed at minimum and maximum SS (SS_min_ and SS_max_) at average PI (dependent on RT, see Table 1) (Figs 7 and 8).

At the L4L5 level, the compression force $F_{L4L5}^{c}$ was similar for SS_min_ and SS_max_ in all four RTs (upper row in Fig 7). The shear force $F_{L4L5}^{s}$ was lower in SS_max_ in all four RTs (lower row in Fig 7). At the L5S1 level, the compression force $F_{L5S1}^{c}$ was moderately lower in SS_max_ in all four RTs (upper row in Fig 8). The shear force $F_{L5S1}^{s}$ was larger in SS_max_ in all four RTs (lower row in Fig 8).

### Muscle forces

**Fig 9:**
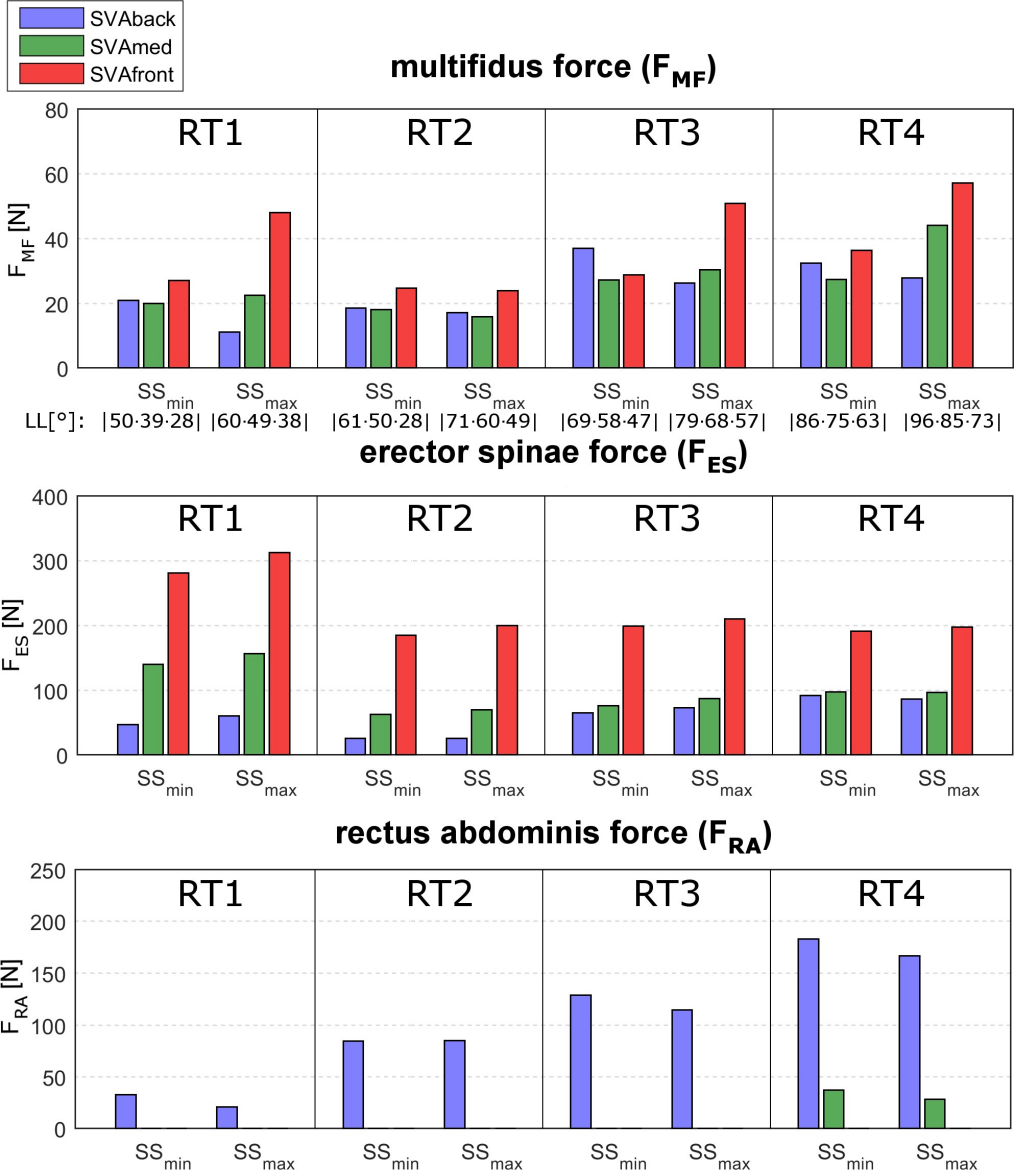
Muscle forces in relation to SS. The muscle forces of the multifidus (F_MF_, upper plot), erector spinae (F_ES_, central plot), and rectus abdominis (F_RA_, lower plot), computed at the minimum and maximum sacral slope values (SS_min_ and SS_max_) in the four lumbar typologies (RT1, RT2, RT3, RT4). SS_min_ and SS_max_ are, respectively, 25° and 35° for both RT1 and RT2, 35° and 45° for RT3, and 45° and 55° for RT4 (Table 1). The results are taken in correspondence to the central pelvic incidence (PI) values for each RT (40° in RT1 and RT2, 50° in RT3, 60° in RT4, Table 1). The three sagittal vertical axis (SVA) conditions interpreting the balanced global alignment (SVAmed) and the backward and frontward imbalanced alignment (SVAback and SVAfront), are presented as green, blue and red, bars, respectively. The lumbar lordosis (LL) is reported as well.

As noted for the intersegmental forces, we observed no relation between PI changes and muscle forces. The multifidus force, F_MF_, was lower in RT1 and RT2, and higher in SVAfront (Fig 9, upper row). The median ranged from 16N to 41N in RT1 and from 17N to 24N in RT2 (Table 3). The force values were higher in RT3 (from 25N to 42N) and RT4 (from 28N to 48N). The F_MF_ was generally higher in SVAfront, and particularly in correspondence with SS_max_ alignments (Fig 9, upper row).

The erector spinae force, F_ES_, was generally higher for SVAfront in all four RTs, with a mild positive linear relation with SS changes (Fig 9, central row). Overall, median values were lower in RT2 (range 25N to 194N, Table 3).

The rectus abdominis was activated only in the SVAback postures in all four RTs and mildly in SVAmed in RT4 (Fig 9, lower row). The muscle force, F_RA_, was increased from RT1 to RT4, and moderately lower in SS_max_. The median force values ranged from 26N to 175N, progressively increasing from RT1 to RT4 (Table 3).

## Discussion

This section begins by discussing the results related to the anatomical parameters in the following order: sagittal vertical axis (SVA) interpreting global sagittal alignment, lumbar typology (RT), and spinopelvic parameters (SS and PI) (Figs 1 and 2). Although lumbar lordosis (LL) was not directly set in the simulated postures, its value was calculated (Figs 7–9). Since the orientations from L5 to L1 were defined as fixed in each RT (Table 1), in order to obtain an LL value dependent on postural change, the lordosis angle was computed between T12 and S1 (Fig 1B). The inclination of T12 was defined as the slope of the vertebral mesh in the thoracic segment, and thus was related to SVA. The slope of S1 matched the SS. Further, LL was indirectly related to RT, as the changes in SS and SVA were dependent on lumbar typology (Table 1).

*SVA–*As expected, a balanced posture (SVAmed) produced lower compression forces in all four RTs than the backward or frontward imbalanced postures (SVAback and SVAfront) (Figs 5 and 6, upper row). The increase in the compression force at L4L5 and L5S1 at SVAfront was particularly pronounced in RT1 (Figs 7 and 8, upper rows). At L4L5, SVAfront also produced greater anterior shear in RT1 and RT2, whereas the shear was increased by SVAback in RT3 and RT4 (Fig 7, lower row). For muscle activation, SVAfront generally led to increased multifidus force, F_MF_, in all four RTs, whereas SVAback did not produce significant alterations (Fig 9, upper row). As expected, the erector spinae and rectus abdominis forces (F_ES_ and F_RA_) were mainly activated in the SVAfront and SVAback postures, respectively (Fig 9, central and lower rows). Indeed, recruitment of the erector spinae (located dorsal to the spine) is necessary to counteract the shifting of trunk weight in postures imbalanced frontward and in balanced alignment as well, albeit with lower forces. Conversely, the rectus abdominis (located frontal in the lumbar region) is activated to counteract postures imbalanced backward. In particular, the rectus abdominis was not activated in either the SVAmed or the SVAfront posture (Table 3). These observations indicate that a frontward imbalanced posture is the one most able to increase loads on the lumbar spine. Our findings also underline the importance of achieving appropriate alignment in spinal reconstruction procedures, e.g., correcting thoracic hyperkyphosis, which is known to cause chronic pain and increase spinal load [29] or correcting hypo-lordosis in reconstruction surgery [30].

*RT–*Lumbar typology RT1 produced larger compression forces at L4L5 (Fig 7, upper row, and Table 2). Both RT1 and RT2 produced lower shear loads (Fig 7, lower row). Furthermore, RT1 was the typology most affected by SVAfront alignment in terms of alterations in compression force. Overall, lower shears were predicted in RT1 with corresponding large SS values (see SS_max_ in lower row of Fig 7).

Conversely, RT1 produced larger shear forces at the L5S1 level. This finding can be explained by the result depicted in Fig 3B. Although the predicted intersegmental loads (F_L5S1_ and F_L4L5_) are similar in modulus and direction, the anterior component of F_L5S1_ (projection of the vector on the anterior axis) is larger than that of F_L4L5_. The difference between the directions of the anterior axes of L5 and S1 is larger in RT1 (43° at SS_max_) than in the other RTs (range 23° to 26°) (Table 1 and Fig 2). RT4 produced larger muscle forces in all the muscles, whereas RT2 produced lower F_MF_ and F_ES_ (Fig 7).

These results highlight the importance of considering lumbar typology when planning the treatment of disorders characterized by alterations in intervertebral lumbar loads. Axial load is associated with the risk of disk bulging and herniation [31–33] and the risk of vertebral fracture in patients with osteoporosis and reduced bone mineral density [34]. RT1 and RT4 warrant particular attention in osteoporosis and in other conditions at increased risk for disc herniation, such as ageing, obesity, and physically demanding work. Moreover, RT1 and RT2 generated lower activation of the multifidus muscle (Fig 9, upper row), the atrophy of which (producing lower forces) is known to be strongly associated with low back pain [35]. Generally, RT3 and RT4 produced increased anterior shear at L4L5 (and RT1 at L5S1), which can be a risk factor of anterior displacement in spondylolisthesis and spondylolysis [36–38].

*SS and PI–*While SS is defined as the slope of the sacral endplate in the sagittal plane (Fig 1), the AnyBody model accounts for bones as simple rigid segments (i.e., spherical or ellipsoidal masses), neglecting their surface shape and properties. As a consequence, the bony mesh surfaces (e.g., sacrum) displayed in the model view have to be considered for visual purposes only, without any mechanical meaning (Fig 3A). In the default standing position of the AnyBody model, the local reference system of the sacrum segment is oriented parallel to the global reference system, but SS visually results approximately 30° (Fig 3A). This orientation was originally set according to anatomic studies [23,24,39]. Because the segment properties (i.e., center of mass, joints, and muscle insertion points) are defined in the local reference system, we simulated the SS changes (along with changes in the corresponding segment properties) by rotating the sacrum segment to account for the SS default value.

Greater SS produced moderately higher compression forces at L4L5 in RT2 and RT3 (Fig 7, upper row) and more consistently affected anterior shear. Indeed, lower and higher shear forces at SS_max_ were noted at L4L5 and L5S1, respectively, in all four RTs (Figs 7 and 8, lower rows). In RT3 and RT4, which are the most common lumbar types in healthy adults [2], postures with greater pelvic retroversion (corresponding to SS_min_ values) produced greater shear loads at L5S1. Accordingly, the degree of sacral inclination should be carefully considered in each RT and in conditions with risk factors of anterior displacement (e.g., spondylolisthesis and spondylolysis). As concerns muscle forces, SS_max_ produced greater F_MF_ in RT4 and moderately greater F_ES_ in RT1, RT2, and RT3 (Fig 9). These results should be interpreted with caution, however, since there is demonstrated evidence for an association between multifidus atrophy and low back pain, while the evidence regarding the erector spinae is conflicting [40].

Surprisingly, changes in PI did not affect compression force at L4L5 (Fig 4C) or at L5S1. This lack of relation with PI was also generally predicted for anterior shear and muscle forces. It is commonly reported and accepted that PI is correlated with the degree of lumbar lordosis (LL). In other words, subjects with a large PI will have a large LL, while those with a small PI have a small LL. A mismatch between PI and LL of less than 10° is targeted in spinal corrective surgery to attain satisfactory spinopelvic alignment [13]. A larger LL is known to cause changes in lumbar loads [7,41,42], whereas PI changes were not found to affect the load distribution in the present study. This finding suggests that although PI and LL are correlated, only the latter parameter can be considered directly responsible for altering spinal load. For example, in the balanced posture (SVAmed), moderately greater compression forces at L4L5 were obtained in RT2, RT3, and RT4 with increased LL (Fig 7, 10° difference between SS_min_ and SS_max_). Furthermore, the PI-LL mismatch has been found as an important parameter associated to increased load in the lumbar spine, specifically in relation to adjacent segment degeneration following fusion surgeries [20,30,43]. Unfortunately, the direct comparison with those studies is not feasible. Indeed, they exploited parameters from pathological subjects or from *in vitro* tests, while the present study has taken into account physiological ranges. Moreover, in the present study the calculation of LL (based on the slope of T12) is potentially affected by assuming thorax as single segment. Future investigations exploiting real patient data and specifically setting LL could elucidate the effects of PI-LL, but also distinguish between the individual contributions of LL and PI.

The present study has several limitations. The AnyBody full-body model represents only the size and weight of an average European male. The stiffness of the intervertebral discs was neglected. The facet joints are not modelled in the default AnyBody model. Nevertheless, they are not expected to have an impact on the loads at the motion segment in the upright position, but rather on the load sharing among the different structures in each motion segment, which is beyond the scope of the present work. The thoracic segment, which was used to obtain the position of C7 and to set SVA (Fig 1), characterizes the twelve thoracic vertebrae and ribcage as a single segment, without allowing the differentiation of further vertebral arrangements. This limitation can cause potential artefacts in the loads at the thoracolumbar level (T12L1), which should be assessed in future studies. The imbalanced postures are modelled by rotating the thorax with respect to L1, instead of being distributed along the different thoracic levels as would be expected physiologically. The present study focused specifically on investigating loads at lower lumbar levels; however, the lumped modelling assumptions for the thorax could have implications at T12L1, which could be carried over and consequently affect loading predictions in the lower lumbar joints as well. Nonetheless, the rigid thorax assumption was demonstrated to affect loading predictions at the upper but not at the lower lumbar levels [44].

Another limitation is the effects of the rotation between the thoracic and the lumbar region (T12L1) and between the lumbar region and the sacrum (L5S1) to obtain the spinopelvic configurations. Indeed, while the lumbar typology (RT) is defined by fixed orientations from L5 to L1 (Table 1), the inclinations of the thorax and the sacrum (in the same RT) varied according to the SVA and SS (obtained by rotating T12L1 and L5S1 joints, respectively). This modelling strategy was necessary, given the absence of subject-specific anatomical data reporting the distribution of rotations between the lumbar and the adjacent spine levels for the specific RT. In principle, however, it could produce large wedge angles at T12L1 and L5S1. In the simulations the extension angle between L5 and S1 ranged from -43° to -13°, which is comparable with that reported in asymptomatic subjects (range -35° to -10°) [45]. Concerning T12L1, since the AnyBody model characterizes the twelve thoracic vertebrae as a single segment, the relative angle between T12 and L1 was obtained by measuring the slope of the mesh of T12 in the thoracic segment (Fig 1B). The angle ranged from -23° to +11°, whereas in asymptomatic subjects it ranged from -7° to +17° [44]. The larger value in extension (-23°), although moderate, can affect the prediction of loads at T12L1. This aspect needs to be better clarified in future investigations, although it is related to the default choice for obtaining orientation of T12. Indeed, as mentioned above, the default AnyBody model does not allow to distribute thoracic rotation along different vertebral levels and constrains the user to orientate the mesh of T12 in the thoracic segment.

A further observation is the dependence of the spinopelvic parameters on each other. The sagittal parameters were varied according to the ranges obtained from the literature and independently one from the other. However, an inherited interdependence is expected in reality, e.g., frontally imbalanced (SVAfront) individuals may not present the lowest SS in a specific RT. Since the values published in the literature describe the distribution in the population, they do not allow to typify the interdependence between the parameters of single individuals. The simulations we evaluated explore all possible configurations among the parameters, including within those typifying interdependency. Nonetheless, real subject-specific data (e.g., from radiographic or CT images) are needed in future model developments to focus on alignments with interdependent parameters.

In conclusion, our findings indicate that changes in global sagittal alignment, lumbar typology, and sacral inclination, but not in pelvic incidence, can affect intervertebral loads in the lumbar spine and spinal muscle activation. Accounting for these variations would be advantageous for clinical evaluation, owing to the relation between altered loads and the risk of disc herniation, vertebral fracture, anterior displacement, and low back pain as well. Musculoskeletal modeling was found to be a valuable biomechanical tool to non-invasively investigate the relation between internal loads and spinopelvic parameters. In order to broaden the extent of the results, future developments will need to assess the relation between loads and anatomical parameters in other poses (e.g., trunk flexion-extension and bending) and dynamic conditions.

## Supporting information

S1 FileResult data.MATLAB data file containing the results reported in the study for the following measurements: $F_{L4L5}^{c}$ and $F_{L4L5}^{s},\;F_{L5S1}^{c}$ and $F_{L5S1}^{s}$, F_MF_, F_ES_, F_RA_, LL. The results are provided in function of RT, SVA, SS and PI changes.(MAT)
